# Regulation of forward and backward locomotion through intersegmental feedback circuits in *Drosophila* larvae

**DOI:** 10.1038/s41467-019-10695-y

**Published:** 2019-06-14

**Authors:** Hiroshi Kohsaka, Maarten F. Zwart, Akira Fushiki, Richard D. Fetter, James W. Truman, Albert Cardona, Akinao Nose

**Affiliations:** 1 Department of Complexity Science and Engineering, Graduate School of Frontier Science, the University of Tokyo, 5-1-5 Kashiwanoha, Kashiwa Chiba, 277-8561 Japan; 2 HHMI Janelia Research Campus, Ashburn, VA 20147 USA; 3 School of Psychology and Neuroscience, University of St Andrews, KY16 9JP Scotland, UK; 4 Departments of Neuroscience and Neurology, Zuckerman Mind Brain Behavior Institute, Columbia University, New York, NY USA; 5 Friday Harbor Laboratories, University of Washington, Friday Harbor, WA 98250 USA; 6 Department of Physiology, Development and Neuroscience, University of Cambridge, Cambridge, CB2 3DY UK; 7 Department of Physics, Graduate School of Science, the University of Tokyo, 7-3-1 Hongo, Bunkyo-ku, Tokyo, 133-0033 Japan

**Keywords:** Neuroscience, Physiology

## Abstract

Animal locomotion requires spatiotemporally coordinated contraction of muscles throughout the body. Here, we investigate how contractions of antagonistic groups of muscles are intersegmentally coordinated during bidirectional crawling of *Drosophila* larvae. We identify two pairs of higher-order premotor excitatory interneurons present in each abdominal neuromere that intersegmentally provide feedback to the adjacent neuromere during motor propagation. The two feedback neuron pairs are differentially active during either forward or backward locomotion but commonly target a group of premotor interneurons that together provide excitatory inputs to transverse muscles and inhibitory inputs to the antagonistic longitudinal muscles. Inhibition of either feedback neuron pair compromises contraction of transverse muscles in a direction-specific manner. Our results suggest that the intersegmental feedback neurons coordinate contraction of synergistic muscles by acting as delay circuits representing the phase lag between segments. The identified circuit architecture also shows how bidirectional motor networks could be economically embedded in the nervous system.

## Abstract (web summary)

Locomotion involves the coordinated contraction of antagonistic muscles. Here, the authors report that in Drosophila larvae a pair of higher-order feedback neurons temporally regulates the intersegmental coordination of contraction of synergistic muscles enabling bidirectional movement.

## Introduction

Axial locomotion, where muscle contractions propagate along a chain of body segments, is a prevalent mode of motor outputs in bilateral animals. This motor pattern includes crawling, swimming, and walking in vertebrates and invertebrates^1–4^. Axial propagation of motor activity is also observed in back muscles during human walking^5^. In general, axial locomotion possesses the following organizational properties. (1) Intersegmental coordination: for the entire body to produce coherent behavioral outputs, motor activities have to propagate along a chain of segments in a phase-coupled manner^1,2,6^. (2) Muscle synergy: to enable efficient recruitment of a large number of muscles in the body during axial or other types of movements, groups of muscles with similar functions are thought to be activated in a modular manner^7–9^. (3) Bidirectionality: while some animals move only in the forward direction, many animals including nematodes, lampreys, and tadpoles, also move in the backward direction by reversing the propagation of motor activity^10–14^.

In spite of intensive studies on each of these regulations in crustacean, leech, lamprey, fish, tadpole, and salamander among others^1–6^, how these regulations are integrated to generate dynamic and orchestrated motor outputs during axial locomotion remains incompletely understood. Neuronal sub-circuits implementing each of these regulations likely converge in a larger circuit to generate a coherent sequence of axial movements. However, little is known about the mechanism of how the intersegmental propagation of motor activity is coupled to coordinated activation and inhibition of synergistic muscles in adjacent segments. Little is also known about the mechanism of how bidirectional sub-circuits generate flows of neural activity that recruit similar groups of muscles in opposite directions. Dedicated subnetworks including both interneurons and motor neurons (MNs) are known to actuate forward and backward locomotion in the unsegmented *Caenorhabditis elegans*^13^. However, such a circuit configuration would increase the number of component neurons and the complexity of the circuits.

*Drosophila* larvae provide an excellent model system for genetically dissecting the neural networks controlling axial locomotion^15,16^. The larvae normally locomote by forward crawling, in which segmental muscle contraction and the corresponding motor activity in the central nervous system (CNS) is propagated from the posterior to anterior body segments^12,17,18^. The larvae also exhibit backward crawling, in which largely the same sets of muscles in each segment are activated, but in the opposite direction from the anterior to posterior. Two major groups of antagonistic muscles are present in each body segment: longitudinal muscles whose activation leads to longitudinal contraction of the segment, and transverse muscles whose activation induces circumferential contraction^12,19^. During both forward and backward locomotion, these two groups of muscles are sequentially contracted in each segment, with the longitudinal muscles being activated first^12^. The relative timing (phase) of the activation of the two groups of muscles is synchronized both intrasegmentally and intersegmentally at varying cycle periods^20^. Furthermore, the phase-constancy is maintained during fictive locomotion in an isolated CNS^18^. These results suggest the presence of central pattern generators that intersegmentally and bidirectionally regulate the coherent activation of the two groups of muscles.

In this study we combined electron microscopy (EM) circuit mapping and functional analyses to identify the neural network that underlies this coordination. The core elements of the network are two bilateral pairs of 2nd order premotor interneurons in each abdominal segment. Each of the paired neurons is specifically activated in forward or backward locomotion, respectively, and through intersegmental feedback provides delayed excitation to the adjacent posterior or anterior neuromere. By linking sub-circuits regulating intersegmental activity propagation and synergistic muscle control in a direction-specific manner, these neurons realize intersegmentally coordinated activation of synergistic muscles. They share a group of premotor interneurons as their postsynaptic partners, which in turn connect with groups of antagonistic muscles in a cooperative manner, demonstrating how neural networks coordinating a bidirectional flow of neural activity could coexist in the CNS.

## Results

### Bidirectional feedback connections in the premotor circuits

**Fig. 1:**
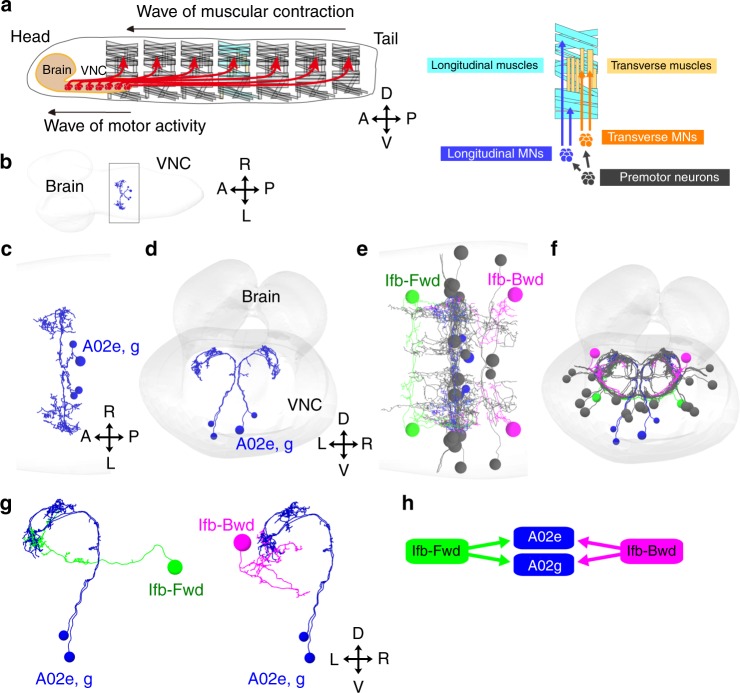
Identification of 2nd order premotor interneurons. Circuit mapping of PMSIs, A02e and A02g and their presynaptic partners from EM. **a** (Left) Schematic of larval muscles and the nervous system. Crawling behavior is generated by the propagation of muscular contraction along the length of the body, which is operated by the propagation neural activity within the central nervous system (VNC: ventral nerve cord). (Right) The body wall muscles are classified as longitudinal and transverse muscles by their orientations. They are innervated by distinct motor neurons, Longitudinal motor neurons (MNs), and Transverse MNs. **b** An image of the entire CNS reconstructed by electron microscopy. The black box indicates the region shown in (**c**). **c**, **d** Dorsal (**c**) and posterior (**d**) views of A02e and A02g (blue) in segment A1. Gray shading represents the outline of the nervous system. **e**, **f** Dorsal (**e**) and posterior (**f**) views of A02e and A02g (blue) and their presynaptic interneurons, Ifb-Fwd (A01d3 in a lineage-based nomenclature, green), Ifb-Bwd (A27k in a lineage-based nomenclature, magenta), and others (gray). **g** Posterior views showing the connection between PMSIs (A02e and g) and the presynaptic partner Ifb-Fwd and Ifb-Bwd. (**h**) A connectivity diagram

**Fig. 2:**
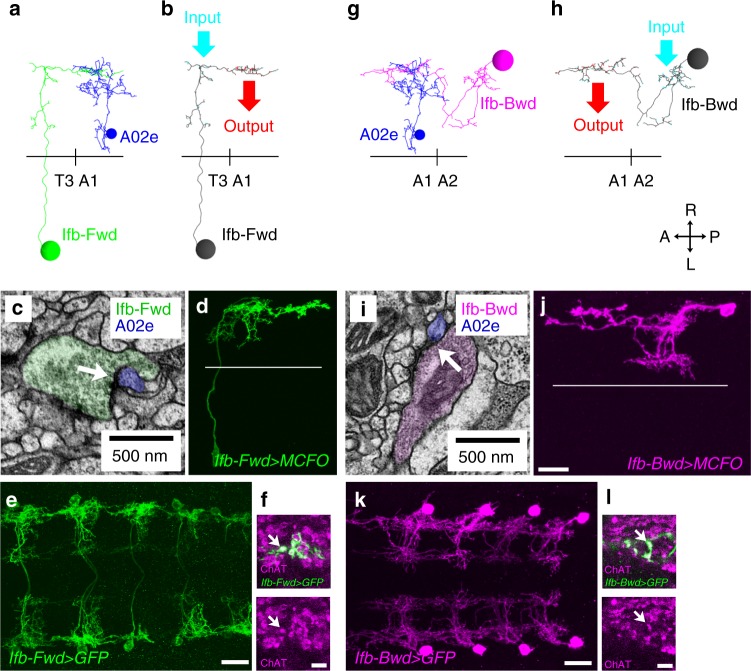
Intersegmental projection of Ifb-Fwd and Ifb-Bwd interneurons. **a**–**f** Analyses of Ifb-Fwd interneuron. **a**–**c** Neuron reconstruction from EM. **a** Dorsal view of the Ifb-Fwd neuron (green) and its target A02e (blue). **b** Location of the synaptic inputs (cyan) and outputs (red) in Ifb-Fwd. **c** A synaptic contact between Ifb-Fwd and A02e (arrow). **d** The morphology of Ifb-Fwd as revealed by clonal analysis in *SS02065>MCFO* (multi-color flip-out). A dorsal view. **e** *SS02065-Gal4* specifically targets Ifb-Fwd. A dorsal view of an *SS02065-Gal4* *>* *mCD8::GFP* CNS stained for GFP. **f** Ifb-Fwd is ChAT-positive. Dorsal view of an *SS02065* *>* *mCD8::GFP* CNS (*n* = 5 larvae). **g**–**l** Analyses of Ifb-Bwd interneuron. **g**–**i** Neuron reconstruction from EM. **g** Dorsal view of the Ifb-Bwd neuron (magenta) and its target A02e (blue). **h** Location of synaptic inputs (cyan) and outputs (red) in Ifb-Bwd. **i** Synaptic contacts between Ifb-Bwd and A02e (arrow). **j** The morphology of Ifb-Bwd as revealed by clonal analysis in *SS026694>MCFO*. A dorsal view. **k** *SS026694-Gal4* specifically targets Ifb-Bwd. A dorsal view of an *SS026694>mCD8::GFP* CNS stained for GFP. **l** Ifb-Bwd is ChAT-positive. Dorsal view of an *SS026694>mCD8::GFP* CNS (*n* = 5 larvae). Black horizontal lines in (**a**, **b**, **g**, **h**) and white horizontal lines in (**d**, **j**) indicate the midlines. Scale bar, 20 µm (**d**, **e**, **j**, **k**) and 5 µm (**f**, **l**)

**Fig. 3:**
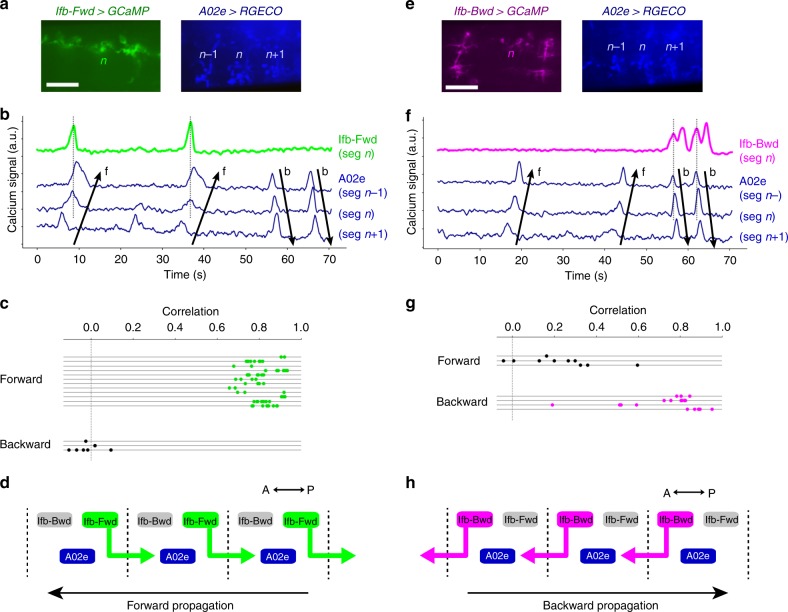
Ifb-Fwd and Ifb-Bwd mediate intersegmental feedback signaling. **a** Temporally stacked images of dual-color calcium imaging (pseudocolored such that *Ifb-Fwd>GCaMP* is green and *A02e>RGECO* is blue). **b** An example of calcium imaging of Ifb-Fwd (green) and A02e (blue). Ifb-Fwd is active during forward (f) but not backward (b) fictive locomotion (64 forward waves and 8 backward waves from 8 larvae). A02e activity was used to monitor wave propagation. **c** Correlation coefficients of calcium signals between Ifb-Fwd and A02e across trials in forward and backward waves. Each horizontal line corresponds to a single trial. **d** Summary of the activity and connectivity of Ifb-Fwd. **e** Temporally stacked images of dual-color calcium imaging (pseudocolored such that *Ifb-Bwd>GCaMP* is magenta and *A02e>RGECO* is blue). **f** An example of calcium imaging of Ifb-Bwd (magenta) and A02e (blue). Ifb-Bwd is active during backward (b) but not forward (f) fictive locomotion (10 forward waves and 19 backward waves from three larvae). **g** Correlation coefficients of calcium signals between Ifb-Bwd and A02e across trials in forward and backward waves. Each horizontal line corresponds to a single trial. **h** Summary of the activity and connectivity of Ifb-Bwd. Scale bar, 40 µm (**a**, **e**)

In each abdominal hemi-segment of *Drosophila* larvae, two major groups of antagonistic muscles, longitudinal muscles, and transverse muscles, are innervated by distinct sets of motor neurons (Fig. 1a). Previous studies identified several groups of segmental premotor interneurons that are active during forward/backward locomotion^15,20–28^, including the PMSIs (*period*-positive median segmental interneurons)^22^. Since PMSIs in each neuromere are sequentially activated in a phasic manner both during forward and backward waves (see below), we reasoned that key regulatory neurons of intersegmental coordination should be present in the common upstream circuits of these neurons. We therefore used reconstructions from a serial section transmission electron microscopy (ssTEM) image data set of an entire first instar larval CNS^29,30^ to investigate the upstream circuits of PMSIs. We first identified in the ssTEM images, two PMSIs, A02e and A02g (Fig. 1b–d), which form synapses to MNs locally in the same segment (Supplementary Fig. 1A–F. For details on the characterization of PMSIs see Supplementary Methods and Supplementary Fig. 1). We then reconstructed the presynaptic neurons of A02e and A02g, and identified Ifb-Fwd and Ifb-Bwd as the only common upstream neurons (Fig. 1e–h and Supplementary Fig. 2), except for the DnB (Down and back) neuron, which had been previously shown to be involved in the regulation of nociceptive escape behaviors^31^. Interestingly, as detailed below, we found that Ifb-Fwd and Ifb-Bwd form symmetric intersegmental feedback circuits (Fig. 2). Furthermore, Ifb-Fwd and Ifb-Bwd were only active during forward and backward motor propagation, respectively (Fig. 3). These observations suggested that these 2nd order premotor interneurons play reciprocal roles in the regulation of bidirectional locomotion.

Ifb-Fwd receives inputs in the dendrites located in the same neuromere as the cell body and projects its axon to the next posterior neuromere to innervate A02e and A02g of that subsequent neuromere, as revealed by the circuit mapping from EM (Fig. 2a–c) and single cell analyses (Fig. 2d, e). Ifb-Fwd expresses a cholinergic marker choline acetyltransferase (ChAT) (Fig. 2f), suggesting that Ifb-Fwd forms excitatory synapses on A02e^32^. Dual-color calcium imaging of Ifb-Fwd and A02e showed that Ifb-Fwd is activated during forward but not backward fictive locomotion (Fig. 3a–c. Correlation coefficients between Ifb-Fwd and A02e are 0.82 ± 0.07 (64 waves) in forward waves and −0.02 ± 0.06 (8 waves) in backward waves (*p* = 2.6 × 10^−42^, the two-sided Student’s *t*-test, *n* = 8 larvae)). We named this neuron Ifb-Fwd (intersegmental feedback during Forward propagation) because the neuron is specifically active during the forward activity propagation (Fig. 3a–c) and sends signals back to the posterior neuromere in the opposite direction to wave propagation (Fig. 2a–f). The circuit configuration and temporal profile of its activity is consistent with Ifb-Fwd having roles in intersegmental coordination of motor activity.

Similar analyses showed that Ifb-Bwd was the counterpart of Ifb-Fwd but during backward locomotion, as follows. Ifb-Bwd received inputs in dendrites present in the same segment as the cell body and projected its axon to the adjacent anterior segment to innervate PMSIs (Fig. 2g-k). Like Ifb-Fwd, Ifb-Bwd expressed ChAT and thus is likely excitatory (Fig. 2l). Ifb-Bwd was activated during backward but not forward locomotion (Fig. 3e–g. Correlation coefficients between Ifb-Bwd and A02e are 0.23 ± 0.18 (10 waves) in forward waves and 0.75 ± 0.18 (19 waves) in backward waves (*p* = 7.3 × 10^−8^, the two-sided Student’s *t*-test, *n* = 3 larvae)). We therefore named it Ifb-Bwd (Intersegmental feedback neuron in Backward propagation). The morphology and activity of Ifb-Fwd and Ifb-Bwd neurons are summarized in Fig. 3d, h.

### Ifb-Fwd and Ifb-Bwd neurons share postsynaptic neurons

**Fig. 4:**
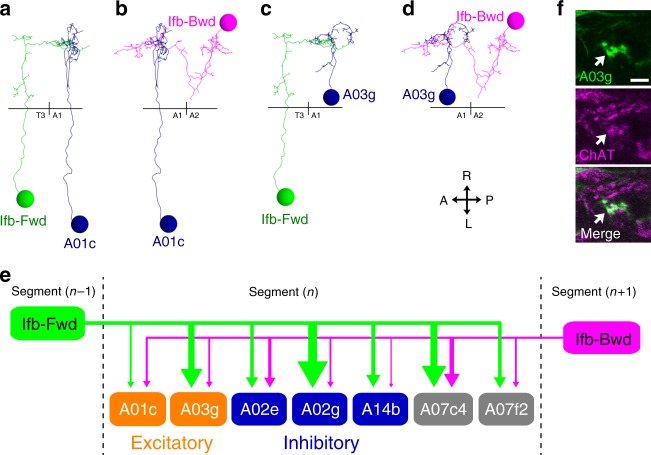
Ifb-Fwd and Ifb-Bwd share postsynaptic target neurons. **a**–**d** Dorsal views of the reconstructed Ifb-Fwd and Ifb-Bwd and their postsynaptic targets A01c and A03g. Black horizontal lines indicate the midlines. **e** Downstream neurons shared by Ifb-Fwd and Ifb-Bwd (excitatory neurons, orange; inhibitory neurons, blue; neurotransmitter unknown, gray). We assigned their neurotransmitter phenotype as follows: A01c was reported to be cholinergic^20^. A14b is putatively GABAergic since it belongs to a lineage of GABAergic neurons^20^. A03g is shown to be cholinergic in (**f**). The neurotransmitter phenotype of the other two neurons (A07c4 and A07f2) could not be tested due to lack of specific Gal4 lines. The widths of arrows indicate synapse numbers. **f** Dorsal view of A03g (*R36G02>mCD8::GFP*) counterstained for ChAT (*n* = 5 larvae). Scale bar, 5 µm

**Fig. 5:**
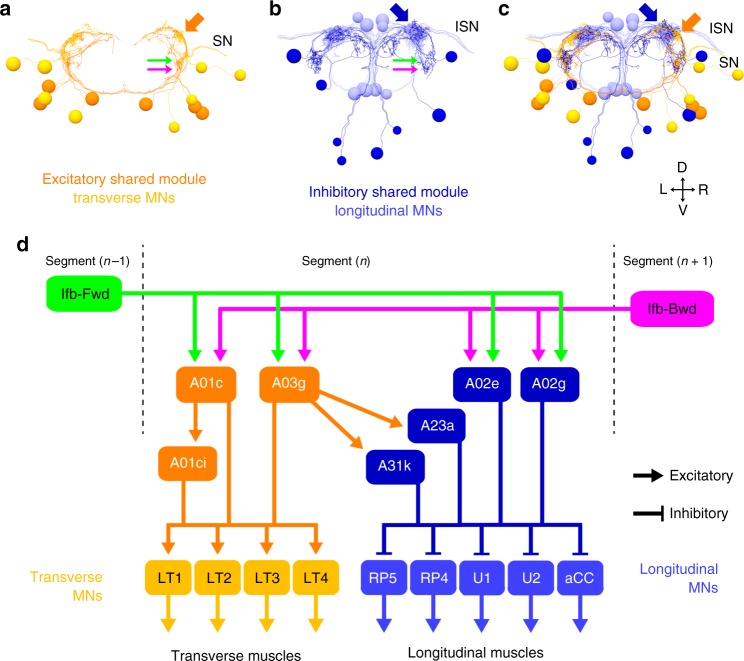
The shared module neurons innervate MNs in a synergistic manner. **a**–**c** Posterior views of the connectivity between the shared module neurons and the target MNs. **a** Excitatory neurons in the module (dark orange) and the target transverse MNs (light orange). **b** Inhibitory neurons in the module (dark blue) and the target longitudinal MNs (light blue). **c** Merged image of (**a**) and (**b**). Arrows indicate the presynaptic sites of excitatory interneurons (dark orange), inhibitory interneurons (dark blue), and postsynaptic sites of the interneurons receiving inputs from Ifb neurons (green and magenta). SN: segmental nerve; ISN: intersegmental nerve. **d** Connectome of the shared module. Excitatory neurons in the module innervate MNs targeting transverse muscles. Meanwhile, inhibitory neurons in the module innervate MNs targeting longitudinal muscles

The circuit configuration of Ifb-Fwd and Ifb-Bwd neurons, where a segment receives feedback signals from the next segment that is activated later in propagation, suggests that these neurons provide delayed excitatory inputs to the adjacent neuromere during wave propagation. We therefore wondered whether these neurons also target other premotor neurons, beyond the PMSIs, to regulate their timely activation during axial locomotion. To test this possibility, we extended the circuit mapping from EM to the downstream circuits of Ifb-Fwd and Ifb-Bwd (Fig. 4a–d). The analyses revealed that these two neurons intersegmentally share a number of postsynaptic target interneurons (Fig. 4e and Supplementary Fig. 3A), including premotor interneurons (Fig. 5d), further supporting the notion that they play reciprocal roles in bidirectional locomotion. Also, Ifb-Fwd and Ifb-Bwd were major sources of inputs to these common target neurons (Supplementary Fig. 3B). We found five common target interneurons in addition to the two glutamatergic and putatively inhibitory PMSIs A02e and A02g described above (and an MN LT1). Two of the five common neurons, A01c and A03g, appeared to be cholinergic and thus excitatory, and one, A14b, was putatively GABAergic and thus inhibitory (Fig. 4e, f). Interestingly, the axons of Ifb-Fwd and Ifb-Bwd overlapped in a restricted region in each neuromere, and the dendritic arbors of the seven common target neurons extended toward this restricted region in various directions (Supplementary Fig. 4; see below), which may represent a functional domain for information processing (see Discussion section).

### The downstream circuits synergistically innervate MNs

Since Ifb-Fwd and Ifb-Bwd neurons likely convey delayed excitation to the neighboring neuromere, they may innervate premotor circuits that activate later-contracting transverse muscles and/or that inhibit earlier-contracting longitudinal muscles. Probing further downstream to MNs in the premotor circuits, we found that both appear to be the case (Fig. 5a–d and Supplementary Fig. 5). The two putative excitatory downstream neurons, A01c and A03g, directly form synapses to MNs that target transverse muscles (hereafter called transverse MNs) (Fig. 5d and Supplementary Fig. 5). A01c also provided putative excitatory inputs via the A01ci neuron (“A01c with ipsilateral dendrites”), which appears to be cholinergic (Supplementary Fig. 6), to transverse MNs. Conversely, inhibitory A02e and A02g neurons directly innervated MNs that target longitudinal muscles (called longitudinal MNs). Furthermore, the excitatory A03g neuron, in addition to directly forming putative excitatory synapses with transverse MNs, provided putative inhibitory inputs via two GABAergic interneurons (A23a and A31k, Supplementary Fig. 6) to longitudinal MNs^12,19^. Thus, the circuit diagram is consistent with the idea that Ifb-Fwd and Ifb-Bwd neurons control muscle movement in a synergistic manner, activating the shared downstream circuits that excite the transverse muscles and inhibiting the antagonistic longitudinal muscles. Previous studies identified A27h and EL as candidate premotor interneurons that excite the longitudinal muscles^23,27^.

### The shared neurons are bidirectionally active

**Fig. 6:**
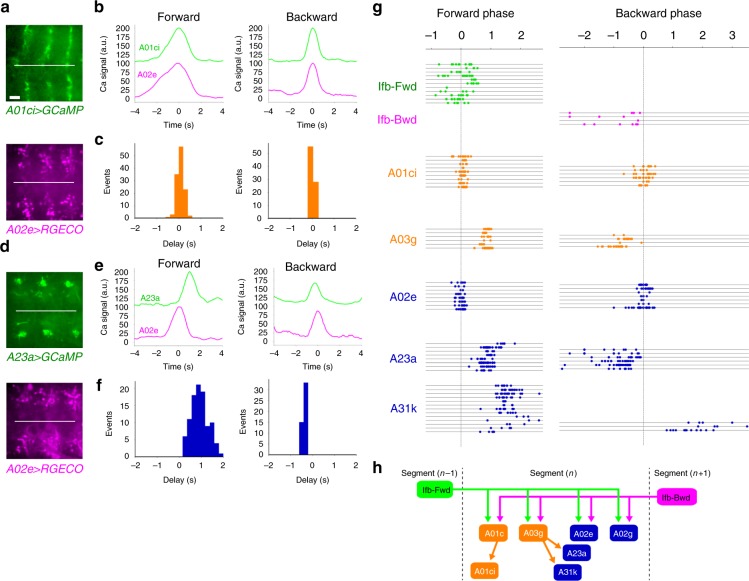
Temporal profiles of the activity of the shared module neurons. **a**–**c** Dual-color calcium imaging of A01ci and A02e. **a** Temporally stacked images of *R75H04>GCaMP* (expressed in A01ci; top) and *R70C01>RGECO1* (expressed in A02e; bottom). **b** Plot of the calcium signal in the presynaptic sites of A01ci (green) and A02e (magenta) in the same neuromere during a forward (left) and backward (right) wave (44 forward waves and 22 backward waves from six larvae). **c** Histogram of the time delay in peak activity. A01ci activity compared with A02e during forward (left) and backward (right) waves. **d**–**f** Dual-color calcium imaging of A23a and A02e. **d** Temporally stacked images of *SS04495>GCaMP* (expressed in A23a; top) and *R70C01>RGECO1* (expressed in A02e; bottom). **e** Plot of the calcium signal from presynaptic sites of A23a (green) and A02e (magenta) (71 forward waves and 23 backward waves from five larvae). **f** Histogram of the time delay as in (**c**). A23a compared with A02e. A23a follows A02e during forward waves, whereas A23a precedes A02e during backward waves. **g** Scatter plot of activity peaks of the shared module interneurons during forward and backward waves. The timing of the activity peaks in different neurons measured by GCaMP were plotted using the A02e activity, which was monitored by RGECO1, as a reference at each motor wave. Note that the spread of the A02e points is not always at zero phase, despite it being the reference, because peaks of GCaMP and RGECO1 signal in the same A02e do not perfectly coincide. Each horizontal line indicates a single trial. Sample size: 3–8 larvae for each neuron. **h** A wiring diagram of the shared module. A part of Fig. 5d is shown. Scale bar, 20 µm (**a**, **d**)

Since Ifb-Fwd and Ifb-Bwd neurons are active in forward and backward waves, respectively, the shared target neurons might be activated in both waves. Convergence of the Ifb neurons on a common set of premotor interneurons which are active in both waves may decrease the number of neurons required to realize the bidirectional locomotion. To test this, we studied the activity of five downstream neurons, A01ci, A03g, A02e, A23a, and A31k, and found that all of them are indeed activated in both forward and backward waves (Fig. 6 and Supplementary Fig. 7). We also studied the time difference between the activity of the downstream neurons, Ifb-Fwd and Ifb-Bwd neurons, and aCC motor neurons using the activity of A02e as the reference time point (in dual-color calcium imaging—see Methods section for details, Fig. 6 and Supplementary Fig. 7). The temporal sequence thus obtained (Fig. 6g) showed that all downstream neurons are activated at a similar time to, or slightly later than, the Ifb neurons, consistent with their being activated by the Ifb neurons during forward and backward waves. This observation suggests that the local circuit of the shared neurons regulates segmental muscle activity under the directionality-specific operation from Ifb neurons. In addition, detailed observation indicates that the phase relationship between the shared neurons depends on the direction of wave propagation (Fig. 6g), suggesting the existence of additional higher-order (2nd or higher) premotor interneurons with direction-specific activity properties. We also noted that the activity of some of the downstream neurons is more closely related to that of other downstream neurons than to others (A01ci/A02e and A03g/A23a, Fig. 6g).

### Contraction of transverse muscles requires Ifb neurons

**Fig. 7:**
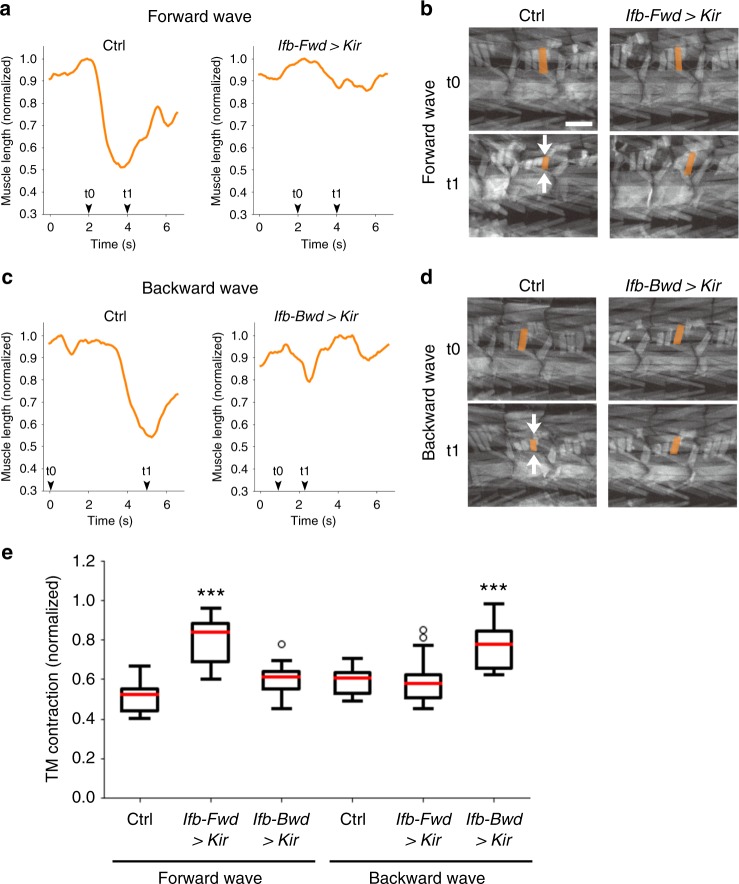
Ifb-Fwd and Ifb-Bwd are required for the contraction of transverse muscles. **a**–**d** Muscle imaging using *mhc-GFP* during forward (**a**, **b**) and backward propagation (**c**, **d**). **a**, **c** The lengths of transverse muscles M23 are plotted. **b**, **d** Images of body wall muscles during wave propagations. White arrows indicate the contraction of the transverse muscle. Note the reduction in the contraction of transverse muscles during forward waves when the activity of Ifb-Fwd is blocked (**a**, **b**) and the reduction in the muscle contraction during backward waves when the activity of Ifb-Bwd is blocked (**c**, **d**). **e** Quantification of the contraction of transverse muscles. Data size: 10–18 waves from 4 to 6 larvae for each group. ***p < 0.005 (Ifb-Fwd > Kir: *p* = 5.6 × 10^−9^; Ifb-Fwd > Kir: *p* = 0.0031 by the two-sided Student’s *t*-test with the Bonferroni correction). See Methods section for statistical analysis. Center line, median; box limits, upper and lower quartiles; whiskers, 1.5× interquartile range; points, outliers. Scale bar, 200 µm (**b**, **d**)

Since likely outputs of Ifb-Fwd and Ifb-Bwd are excitation of transverse muscles and inhibition of longitudinal muscles (Fig. 5d), Ifb neurons may regulate contraction and relaxation of these muscles. Indeed, we observed decreased contraction in transverse muscles when the activity of Ifb neurons was blocked. We blocked the activity of Ifb neurons by expression of the rectified potassium channel Kir and studied the effect on the dynamics of muscle contraction that occurred during motor wave propagation in open-filleted larvae (muscle contractions imaged by using the *mhc-GFP* transgene, Fig. 7). In control larvae, transverse muscles contracted both during forward and backward waves, which is consistent with previous studies^12^. When the activity of Ifb-Fwd is blocked, contraction of the transverse muscles was significantly reduced during forward but not backward waves (Fig. 7a, b and e). Thus, Ifb-Fwd is required for proper contraction of transverse muscles during forward motor propagation. Conversely, when the activity of Ifb-Bwd was blocked, contraction of transverse muscles was compromised in backward but not forward waves (Fig. 7c–e). These observations indicate that Ifb-Fwd and Ifb-Bwd neurons together are critical for delayed activation of the transverse muscles in bidirectional axial locomotion.

We also temporally inhibited the transmission of Ifb-Fwd neurons and studied the effects on larval locomotion. The speed of larval locomotion was significantly decreased (Supplementary Fig. 8; *Ifb-Fwd>shi*, 0.51 ± 0.01 s per wave (*n* = 22); driver control, 0.44 ± 0.02 s per wave (*n* = 20); effector control, 0.38 ± 0.01 s per wave (*n* = 16), *p* = 0.003 (driver control) and *p* = 8 × 10^−8^ (effector control) by the two-sided Student’s *t*-test with the Bonferroni correction), suggesting that Ifb-Fwd neurons contribute to the regulation of axial wave propagation.

We next asked whether activation of Ifb neurons or shared downstream neurons is sufficient to drive contraction of transverse muscles. We activated these neurons by optogenetics and found that activation of Ifb-Fwd or Ifb-Bwd did not induce any muscle movement (Ifb-Fwd: six trials; Ifb-Bwd: five trials). However, we found that activation of the downstream A01ci induces contraction of transverse but not longitudinal muscles (Supplementary Fig. 9). This is consistent with the idea that activation of the excitatory neurons in the downstream circuits by cooperated activation of Ifb neurons and other 2nd order premotor interneurons induces contraction of transverse muscles. Taken together, our results suggest that the intersegmental feedback neurons Ifb-Fwd and Ifb-Bwd mediate delayed activation of transverse muscles via shared downstream circuits and additional synaptic steps in the feedback path.

## Discussion

Sequential recruitment of distinct groups of segmental muscles is commonly seen in axial locomotion^1,2,6^. One previously proposed mechanism for the sequential recruitment is regulation by local circuits present within each segmental unit. For instance, during leech swimming, antiphasic contraction of dorsal and longitudinal muscles is regulated in part by central oscillator circuits present in each ganglion^2,3^. In *Drosophila* larvae, a local inhibitory interneuron (iIN1) regulates delayed contraction of transverse muscles by preventing their precocious activation and thus by acting as an intrasegmental delay circuit^20^. In this study, we revealed a novel mechanism for the sequential muscle recruitment in which intersegmental feedback neurons deliver delayed excitation on premotor circuits. Thus, in *Drosophila* larvae, two delay circuits, one intrasegmental and inhibitory and the other intersegmental and excitatory, function in a complementary manner to ensure timely contraction of the transverse muscles.

**Fig. 8:**
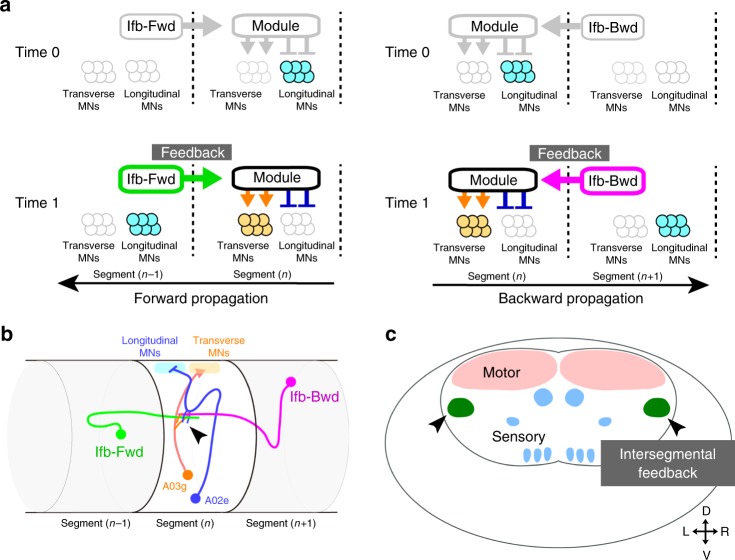
Summary of intersegmental feedback network in motor circuits. **a** Model of the roles of intersegmental feedback neurons Ifb-Fwd (left) and Ifb-Bwd (right) in transforming the progression of a wave front to the intersegmental delay in forward (left) and backward (right) propagations, respectively. **b** Schematic of connectivity from intersegmental feedback neurons (Ifb-Fwd and Ifb-Bwd) through the shared module (A02e and A03g, as examples) to MN synergy (longitudinal MNs and transverse MNs) in the nerve cord. Ifb neurons and the shared neurons form synaptic contacts at a centrolateral region of the neuropil (arrowhead). **c** Schematic of functional domains within the neuropil. Intersegmental feedback signals are conveyed at the centrolateral regions (arrowheads)

The intersegmental delay circuits feedback a signal in the opposite direction to wave propagation, from the wave front to the adjacent segment behind the front. This circuit configuration would enable simultaneous activation of transverse muscles in one segment (via the delay circuit) and longitudinal muscles in the forward adjacent segment (by the wave front) regardless of the speed of wave propagation. The circuit configuration would also maintain the relative timing (phase) of the two groups of muscles: in many axial motions including larval crawling, phase of individual muscle movements is maintained independent of the axial speed, in order to generate functional motor outputs^1–6,33–36^. As shown in Fig. 8a, each segment receives excitatory inputs at two distinct time points: at the arrival of the wave front (time 0) and at a later phase via the intersegmental delay circuits (time 1). Since the time delay (between time 0 and time 1) depends on wave propagation along the neighboring segments and thus scales with the cycle period (or speed), this circuit configuration phase-locks the time delay between the two groups of muscles (Fig. 8a).

Calcium imaging of Ifb and the downstream neurons revealed complex yet precise control of the activity of each premotor interneuron (Fig. 6g) and suggests for the presence of intricate segmental networks. While previous studies identified several interneurons that are implicated in the control of motor activity and/or wave propagation^15,20–28^, the segmental CPG mechanism of rhythm generation remains largely unknown. Further investigation of the circuits upstream and downstream of Ifb neurons will shed light on the nature of the CPG mechanism.

The bidirectional information flow mediated by the intersegmental feedback neurons, Ifb-Fwd and Ifb-Bwd, converge in the immediate downstream circuits on a group of premotor interneurons. This circuit configuration would greatly decrease the number of neurons required to regulate bidirectional locomotion and simplifies the neural wiring as compared with dedicated subnetworks for each direction. Thus, our results suggested an efficient and economical mechanism for implementing bidirectional propagations in the nervous system.

Is a similar strategy used for the bidirectional feedforward circuits? Previous work identified a chain of intersegmental neural connections implicated in feedforward activity propagation during forward locomotion^23^. The key element of the circuits is the intersegmental interneuron A27h, which like the Ifb-Fwd neurons extends its axon into the neighboring segment and is active only during forward motor propagation. Identifying the counterpart of A27h during backward locomotion and studying whether the downstream circuits are also shared will determine whether and how intersegmental inputs are converged in the bidirectional feedforward circuits.

The shared targets of the bidirectional feedback neurons include two premotor interneurons, A01c and A03g, that directly and indirectly provide putative excitatory drive to MNs targeting transverse muscles. When the activity of the delay circuit neurons was blocked, the transverse muscles LT1-4 failed to contract (Fig. 7a–e). Furthermore, activation of A01ci, one of the interneurons in the excitatory pathways, is sufficient to contract these muscles (Supplementary Fig. 8). These observations are consistent with the idea that the feedback neurons act via the common excitatory pathways to mediate synergistic activation of the group of muscles with similar function. The feedback neurons also provide putative inhibitory inputs on the antagonistic longitudinal muscles via the common target neurons, A02e, A02g, and A03g. Thus, the circuit configuration allows simultaneous activation of a group of muscles and inhibition of the antagonistic group of muscles in an intersegmentally coordinated manner. Such circuit configurations may also underlie synergistic motor control observed in other systems (e.g., muscle synergies^7–9^).

All shared target interneurons arborize dendrites in a centrolateral region of the neuropil and receive inputs from the bidirectional feedback neurons in the same domain (arrowheads in Fig. 8b, c). The domain is distinct from the dorsal motor domain and ventral somatosensory domain, thus identifying a novel neuropil compartment where circuits responsible for higher-order motor regulation are integrated. The common target neurons are derived from different progenitor cells (neuroblasts; A01c and A01ci from NB1-2^37^, A02e and A02g from NB 2-1^37^, A03g from NB7-1, and A23a from NB7-4^37^) and their cell bodies are scattered across the CNS. Therefore, there must be intricate axon and dendrite guidance mechanisms independent of progenitor identity^38^ that incorporate these neurons into the integrated circuits and thus enable the higher-order motor coordination. Our circuit mapping and analysis revealed exquisitely and economically constructed neural circuits for motor coordination, and provides a starting point for future studies of how these circuits self-assemble during embryonic development and have appeared through evolution.

## Methods

### *Drosophila* melanogaster strains

All animals were raised on standard cornmeal-based food. Prior to optogenetic experiments, yeast paste containing all-*trans* retinal (1 mM) was fed for 1–2 days. We used the following fly lines: *period-Gal4*^22,39^ (Bloomington #7127), the split-Gal4 drivers^40,41^ *SS02065-Gal4* (*R32C05-p65ADzp* in attP40, *R38E10-ZpGDBD* in attP2), *SS26694-Gal4* (*VT020818-p65ADzp* in attP40, *R91E03-ZpGDBD* in attP2), *SS01817-Gal4* (*R09A07-p65ADzp* in attP40, *R70C09-ZpGDBD* in attP2), *SS04495-Gal4* (*R41G07-p65ADzp* in attP40, *R78F07-ZpGDBD* in attP2) and *SS04399-Gal4* (*R20A03-p65ADzp* in attP40, *R93B07-ZpGDBD* in attP2), *capaR-Gal4* (Texada M.J. and Truman J.W., unpublished), the Rubin-collection^42^ *R75H04-Gal4* (Bloomington #39909) and *R36G02-Gal4* (Bloomington #49939), *eve[RRa-F]-Gal4*^43^, MCFO (multi-color flip-out) lines^44^, *UAS-mCD8::GFP*^45^ (Bloomington #32194), *UAS-GCaMP5G*^46^ (Bloomington #42037), *UAS-KCNJ2:GFP*^47^ (Bloomington #6596), *UAS-shibire*^ts^^48^, *UAS-Syt::HA*^49^, *UAS-ChR2::T159C*^23^, *LexAop-RGECO1*^22^, and *mhc-GFP*^50^.

### Generation of transgenic lines

The codon usage in a sequence encoding a fusion protein of CD4 and GCaMP6f was optimized for *Drosophila melanogaster* (Bio Basic Inc, Canada). For generating *UAS-CD4::GCaMP6f* transgene, the DNA fragment of *CD4::GCaMP6f* gene^51,52^ with KpnI site and translation enhancer Syn21^53^ at the 5′ end and XbaI site at the 3′ end was cloned into pJFRC28-10xUAS-IVS-GFP-p10 plasmid^53^. The transgene was inserted at *attP40* and *VK00005* loci (BestGene Inc., USA) to generate *UAS-CD4::GCaMP6f* transgenic lines. To generate *R70C01-lexA* line, the enhancer sequence of *R70C01-Gal4* was cloned into pBPLexA::p65Uw and pBPnlsLexA::GADflUw plasmids^40^. The transgenic lines were generated in the *attP40* and *VK00027* loci (BestGene Inc., USA).

### Reconstruction of motor circuits using ssTEM data

Acquisition and analysis of ssTEM data is described in Ohyama et al., Zwart et al., and Schneider-Mizell et al.^20,29,30^. PMSIs in the A1 segment were identified and reconstructed within the ssTEM volume based on the axonal projection patterns and dendritic branches (Supplementary Fig. 1). All synapses onto A02e and A02g were annotated and used to find all presynaptic partners including Ifb-Fwd and Ifb-Bwd. Then, all synapses onto or from Ifb-Fwd and Ifb-Bwd were annotated and used to identify presynaptic and postsynaptic neurons of Ifb-Fwd and Ifb-Bwd. All synapses from A02e, A02g, A01ci, A03g, A23a, and A31k were annotated and used to identify the downstream of these interneurons.

### Immunohistochemistry

Immunohistochemistry was performed in the 3rd instar CNS by fixation with 3.7% formaldehyde for 30 min at room temperature, washing with 0.2% Triton X-100 in PBS for 30 min at room temperature, blocking with normal goat serum for 30 min at room temperature and staining with the following antibodies at 4 °C overnight. Primary antibodies: rabbit or guinea pig anti-GFP (Frontier science, Af2020 and Af1180, 1:1000), mouse anti-Fas2^54^ (DSHB #1D4, 1:10), rabbit anti-vGluT^55^ (1:1000), mouse anti-ChAT^56^ (DSHB #4B1, 1:50), rabbit anti-GABA^57^ (Sigma #A2052, 1:500), rabbit anti-DsRed (Clontech #632496, 1:500), and rabbit anti-HA (Cell Signaling Technology #3724 s, 1:500). Secondary antibodies: Alexa488 or Cy3-conjugated goat anti-rabbit IgG, Alexa488-conjugated goat anti-guinea pig IgG and Alexa555 or Cy5-conjugated goat anti-mouse IgG (Invitrogen, 1:500).

### Calcium imaging

Third-instar larvae were dissected by microscissors^22^. The CNS was isolated by cutting off the nerves, tracheae and imaginal discs, and then placed in a drop of saline on a MAS-coated slide glass (Matsunami glass, Japan). The saline was replaced with TES buffer (TES 5 mM, NaCl 135 mM, KCl 5 mM, MgCl_2_ 4 mM, CaCl_2_ 2 mM, sucrose 36 mM; pH = 7.15). The fluorescence of GCaMP6f was detected by a spin-disk confocal unit (CSU21, Yokogawa, Japan) and an EMCCD camera (iXon, Andor Technology, Germany) attached to an upright microscope, Axioskop2 FS (Zeiss, Germany) with a 60x water immersion objective lens. Dual-color calcium imaging of GCaMP6f and R-GECO1 was carried out by using a dual view system (CSU-DV, Solution Systems, Japan)^22^. For detecting peaks of the calcium imaging data in Fig. 6 and Supplementary Fig. 7, the original raw data were smoothed by the Savitzuky-Golay filter. Data analyses were conducted by ImageJ (NIH, USA) and Python3 scripts. For evaluating correlated activity between Ifb neurons and A02e for each wave (Fig. 3), we calculated the correlation coefficient between A02e activity (which was fit to the Gaussian function) and a raw trace of Ifb neuron activity by Python3.

### Larval locomotion assay

To conditionally inhibit neural activity using temperature-sensitive dynamin mutant Shibire^ts^^48^, an agar plate (about 9 cm in diameter) was held at 32 °C on a heat plate (ThermoPlate, Tokai Hit, Japan). Third-instar wandering larvae were gently washed in deionized water and lifted on the agar plate for acclimation (60 sec). The larvae were then videotaped under stereoscopic microscopy (SZX16, Olympus, Japan). The speed of the propagation was measured manually using ImageJ software (NIH).

### Statistical analysis

In Fig. 7e, we performed a normality test by using the Shapiro-Wilk test, with *a* = 0.05. Since data in Fig. 7e passed this test, we performed the two-sided Student’s *t*-test with the Bonferroni correction for comparisons between multiple groups.

### Analysis of muscle activity

We pinned down third-instar larvae on a Sylgard-coated dish, cut along the dorsal side, and removed the internal tissues, while leaving the CNS and all neuromuscular contacts intact. These larva fillets, expressing GFP in muscles, were illuminated with the blue light of a mercury lamp (X-Cite, Olympus, Japan) and monitored for spontaneous forward and backward waves in TES buffer (see Calcium Imaging section), which were recorded by an EMCCD camera (iXon, Andor Technology, Germany).

In the case of optogenetic stimulation, the same light source was used to activate ChR2 expressed in interneurons. The length of transverse muscles was traced manually using ImageJ (NIH, USA). The length of longitudinal muscles was measured by a custom Python3 script. In Fig. 7e, the minimum lengths of transverse muscles (TMs) during wave propagations were normalized by the lengths of relaxed TMs.

### Reporting summary

Further information on research design is available in the Nature Research Reporting Summary linked to this article.

## Supplementary information

Supplementary Information

Reporting Summary

## Data Availability

The data that support the findings of this study are available from the corresponding authors upon reasonable request.

## Code availability

The code used for the analyses are available from the corresponding authors upon reasonable request.
