# Supplementary material for: Regulation of forward and backward locomotion through intersegmental feedback circuits in Drosophila larvae
Source: Nat Commun. 2019 Jun 14;10:2654. doi: 10.1038/s41467-019-10695-y (PMC6572865; doi:10.1038/s41467-019-10695-y)
Supplement: Supplementary file 1 — Supplementary Information [file 41467_2019_10695_MOESM1_ESM.pdf]

Supplementary Information

Regulation of forward and backward locomotion through intersegmental feedback circuits in *Drosophila* larvae

Kohsaka et al.

Supplementary Methods

Circuit mapping of PMSIs from EM

We first reconstructed candidate PMSI neurons in the ssTEM images and compared them to confocal images obtained by clonal analysis (Supplementary Fig. 1A) of each of the ten PMSIs in each hemi-neuromere. The same set of interneurons (termed A02a to A02j [1]) was identified to be present in the neuron reconstruction from EM, based on the soma location, neurite arborization, and position of presynaptic sites (Supplementary Fig. 1A). In the neuron reconstruction from EM, no further neurons possessing axon projections characteristic of PMSIs appeared, suggesting that there are no missing or unanalyzed PMSI neurons. Of the ten PMSIs, we focused our analysis on two, A02e and A02g, which form synapses to MNs locally in the same segment (Supplementary Fig. 1B – 1F, and data not shown) and receive few afferent sensory inputs (Supplementary Fig. 1G) and thus are largely innervated by the 2nd order premotor neurons. We excluded the other PMSIs from further analysis since they form synapses to MNs intersegmentally (A02i and j), and do not form synapses to MNs (A02c) or receive major inputs from sensory neurons (A02a, b, d, f and h) (Supplementary Fig. 1).

We next reconstructed the presynaptic neurons of A02e and A02g, and identified eleven and seven major presynaptic interneurons respectively (Fig. 1E, 1F and Supplementary Fig. 2). We reasoned that key upstream neurons regulating multiple 1st order premotor neurons would innervate both A02e and A02g. We therefore focused on two common presynaptic neurons, Ifb-Fwd (A01d3 in a lineage-based nomenclature) and Ifb-Bwd (A27k in a lineage-based nomenclature) (Fig. 1G and 1H), in this study. The other common presynaptic neuron, DnB (Down and back [2]) was excluded because it was not active during fictive locomotion and thus is unlikely to be involved in its regulation (forward waves: n=5 events; backward waves: n=8 events).

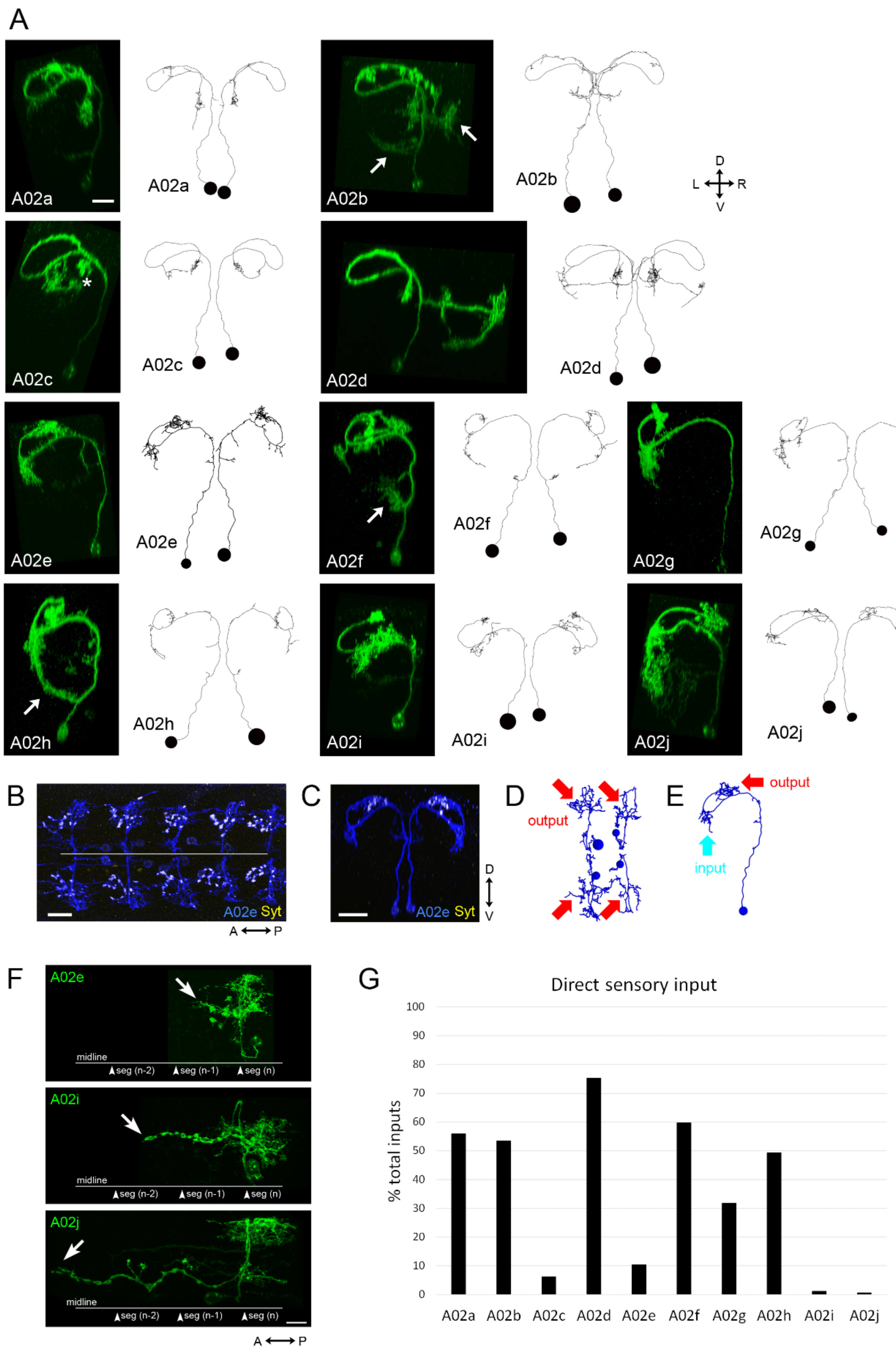

A
A02a
A02b
A02c
A02d
A02e
A02f
A02g
A02h
A02i
A02j
D
L
R
V
B
A02e Syt
A
P
C
D
V
D
output
E
output
input
F
A02e
midline
seg (n-2)
seg (n-1)
seg (n)
A02i
A02j
G
Direct sensory input
% total inputs
100
90
80
70
60
50
40
30
20
10
0

Supplementary Figure 1. Single cell analysis of PMSIs

(A) Single cell images of PMSIs (A02a – A02j) by light microscope (left), and neuron reconstruction from EM in posterior view. The light microscope images were obtained by clonal analyses using *period-Gal4 > MCFO*. Putative dendrite location receiving direct sensory projections is shown by arrows. The asterisk shows the presynaptic terminals of A02c, where few motor neuronal dendrites are targeted, indicating that A02c does not innervate MNs directly. (B and C) Dorsal (B) and posterior (C) views of *SS01817 > Syt-HA* CNS showing the neurites (blue) and presynaptic sites (yellow) of a PMSI, A02e (n=2 larvae). (D and E) Dorsal (D) and posterior (E) views of the location of synaptic inputs and outputs in A02e. Note that the location of the output sites is consistent with that of the presynaptic marker in (B) and (C). (F) Dorsal views of light microscope images of A02e, A02i and A02j. The location of TP1 projection [3] in each segment is shown by an arrowhead with relative segmental number as segmental position marker. The tips of presynaptic terminals shown by arrows indicate that A02i and A02j target neighboring segments. (G) The fraction of direct inputs onto PMSIs (A02a - A02j) from somatosensory neurons in the body wall. The fractions are small in A02c, e, g, I and j. Scale bar, 10 µm (A and F) and 20 µm (B and C).

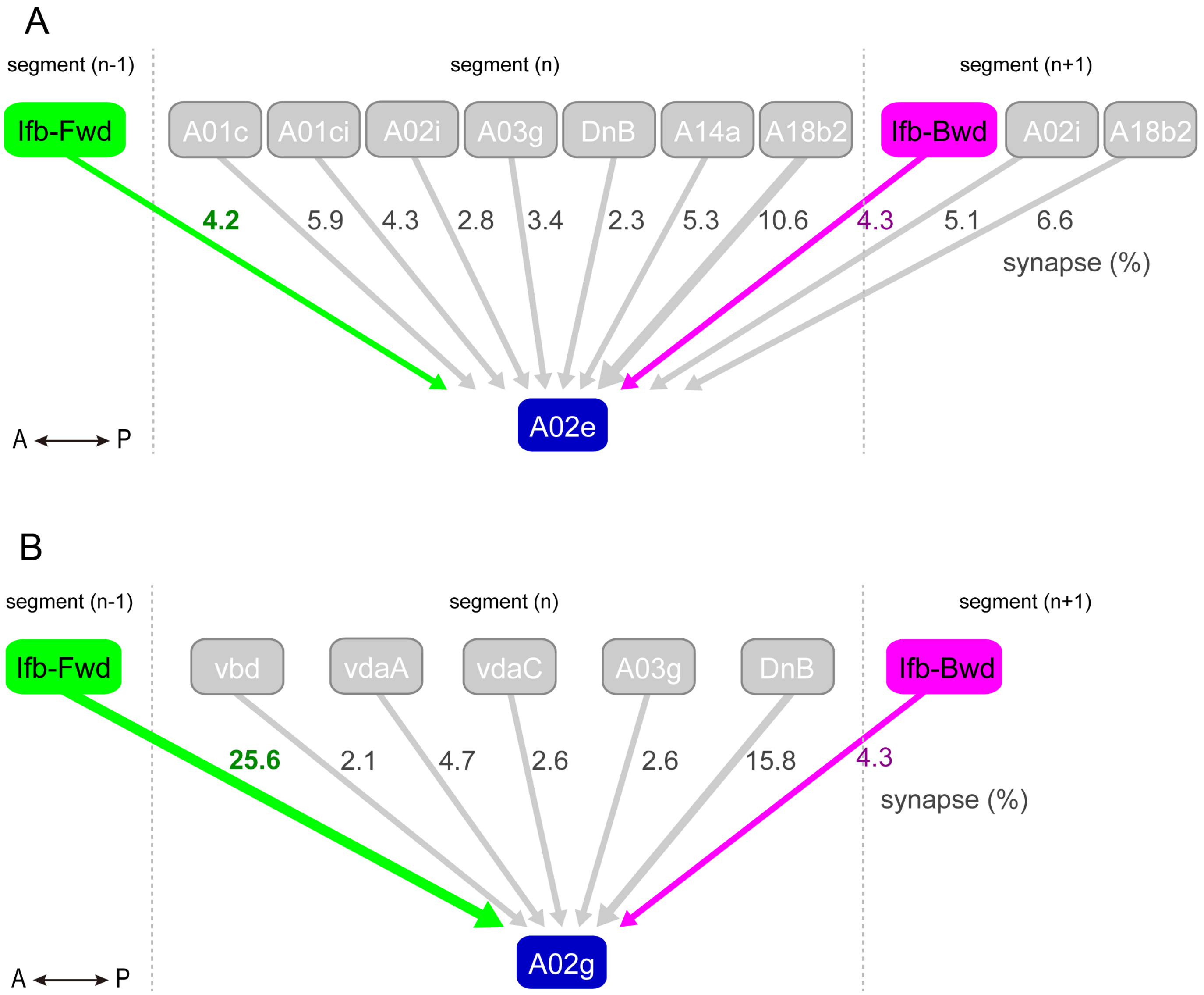

Supplementary Figure 2. Circuit mapping of presynaptic partners of PMSIs, A02e and A02g from EM
Major presynaptic partners (occupying more than 2% of total presynapses) of A02e (A) and A02g (B) are shown. Numbers on arrows indicate the fraction of synapse numbers to total synapses onto A02e (A) and A02g (B).

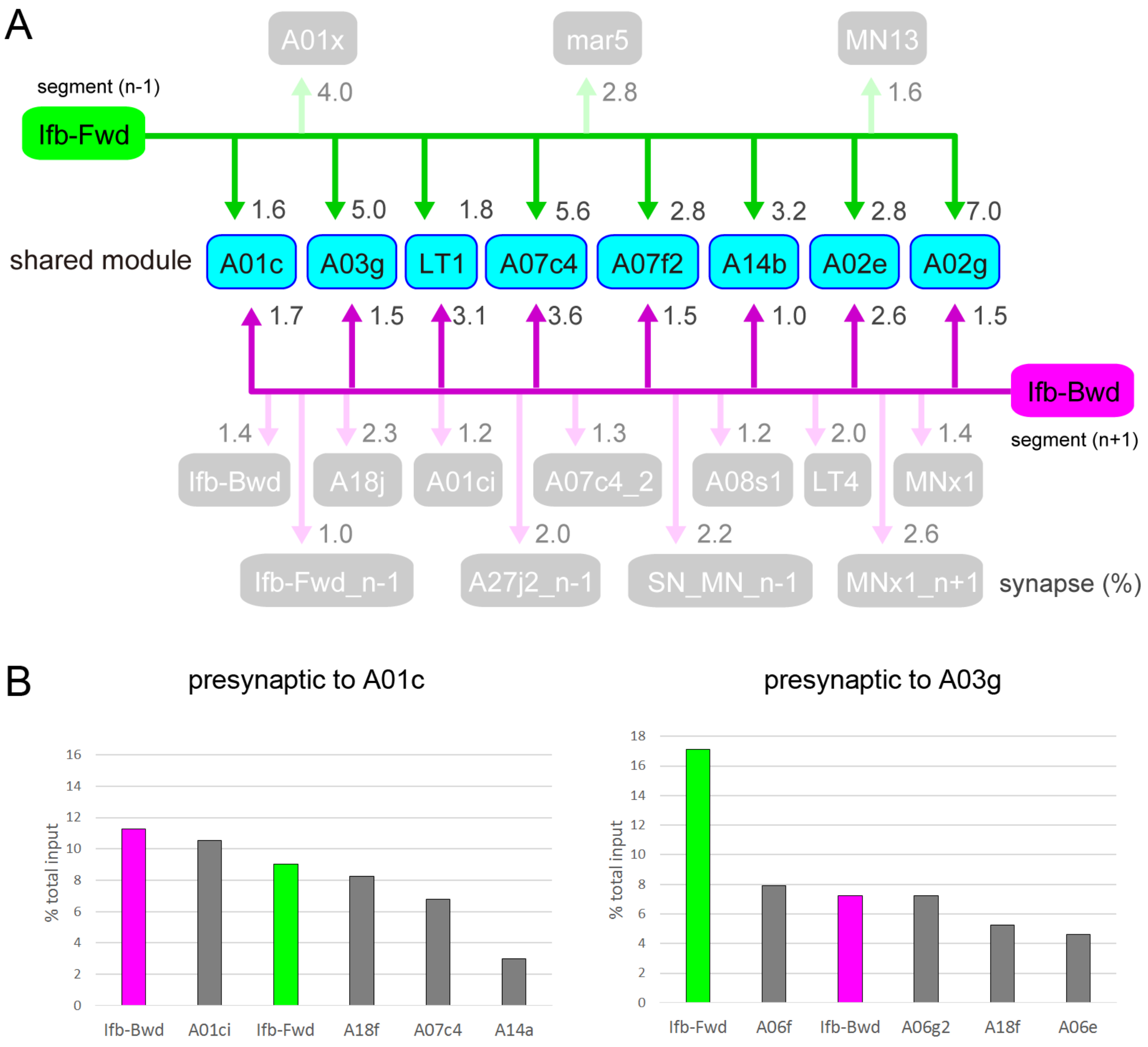

Supplementary Figure 3. Circuit mapping of postsynaptic partners of Ifb-Fwd and Ifb-Bwd from EM
(A) Major postsynaptic partners (occupying more than 1% of total postsynapses) of Ifb-Fwd and Ifb-Bwd are shown. Numbers on arrows indicate the fraction of synapse numbers to total synapses from Ifb-Fwd (on green arrows) and Ifb-Bwd (on purple arrows). Several postsynaptic neurons reside in the neighboring segments indicated by “n-1” (anterior next segment) or “n+1” (posterior next segment). The other neurons are located at segment n. Shared neurons are shown in blue. (B) The fraction of inputs onto A01c (left) and A03g (right). The top 6 presynaptic partners to A01c and A03g are shown.

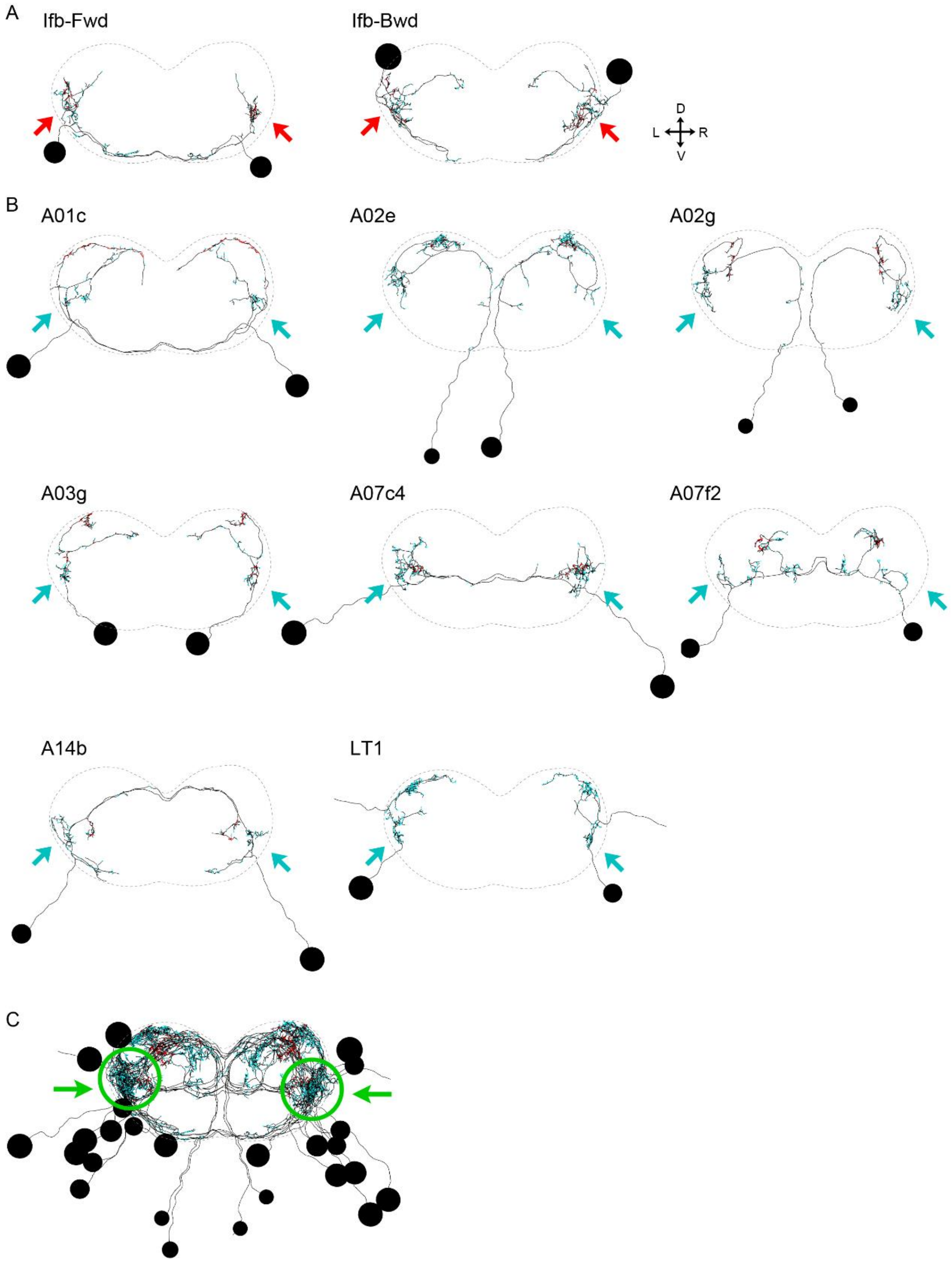

Supplementary Figure 4. Reconstructed images of the Ifb neurons and the shared module neurons
(A and B) Posterior views of the reconstructed Ifb-Fwd and Ifb-Bwd (A) and the shared module neurons (B). Arrows indicate the presynaptic sites of Ifb neurons (red) and the postsynaptic sites of the shared module neurons (blue). (C) Overlay image of Ifb neurons (A) and the shared module neurons (B). The presynaptic sites of Ifb neurons and the postsynaptic sites of the shared module neurons are accumulated at the centrolateral region of the neuropil (green open circles and arrows).

		A01c	A01ci	A03g	A02e	A02g	A23a	A31k	
Longitudinal-MNs	aCC						24	49	
	U1						45	60	
	U2				9		38	93	
	U5				11			23	
	ISN-MN(1)							27	
	ISN-MN(2)			16				37	
	MN20	22							
	RP2							47	
	RP3				8				
	RP5				15	6			
	RP1				32	32			
	RP4				22	34			
	MN27	14	11	8					
	LO1						15		
Transverse-MNs	LT1	49	26	19					
	LT2	35	15	12					
	LT3	37	32	8					Ach (excitatory)
	LT4	29	17	8					GABA (inhibitory)
	MN18	15		8					Glu (inhibitory)
INs	A01c		14						
	A01ci	17							
	A02e	23	29	20					
	A02g			9					
	A03a5			9					
	A03d/e							6	
	A05k							16	
	A06e			8					
	A06f			12					
	A18j	2	13						
	A19f	3	6						
	A19l			26					
	A23a			8					
	A27j		4						
	A31k			62					
	DL2						14		
		A01c	A01ci	A03g	A02e	A02g	A23a	A31k	

Supplementary Figure 5. Sheet of downstream connectivity of the shared module.
The numbers of postsynaptic sites of the shared module (A01c, A01ci, A03g, A02e, A02g, A23a, and A31k) are shown in each column. The downstream MNs are grouped by the class of target muscles (longitudinal or transverse). Cells in the spreadsheet indicating the connection between the shared neurons and MNs are colored based on the neurotransmitter of the shared neurons.

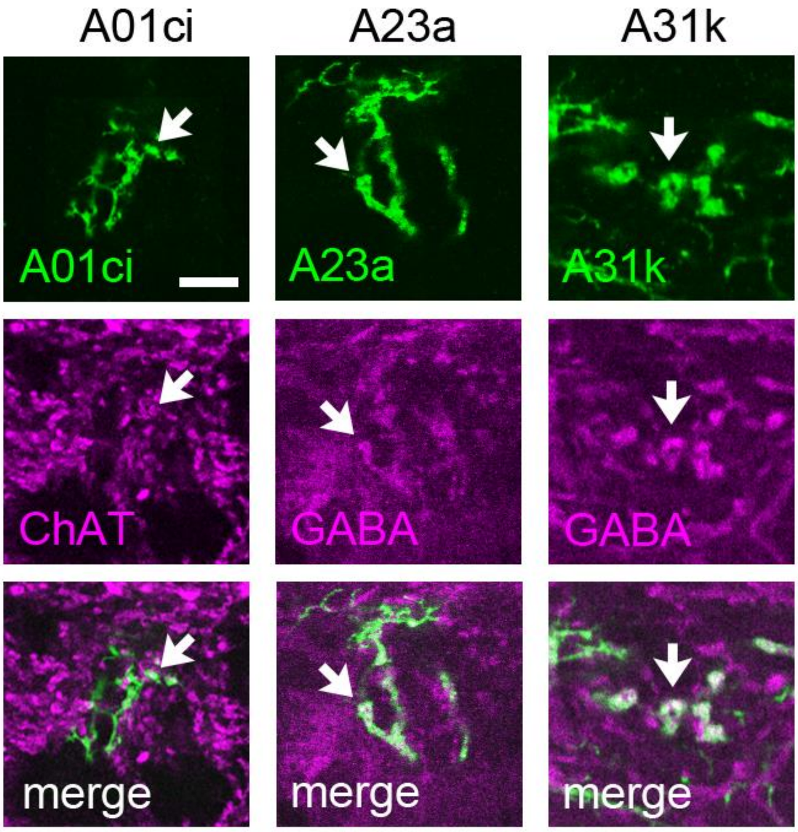

Supplementary Figure 6. Neurotransmitters of the shared neurons.

Dorsal view of A01ci (left; *R75H04 > mCD8::GFP*), A23a (middle; *SS04495 > mCD8::GFP*) and A31k (right; *SS04399 > mCD8::GFP*) counterstained for ChAT (left) and GABA (middle and right) (n=2-4 larvae for each neuron). Scale bar, 5 µm.

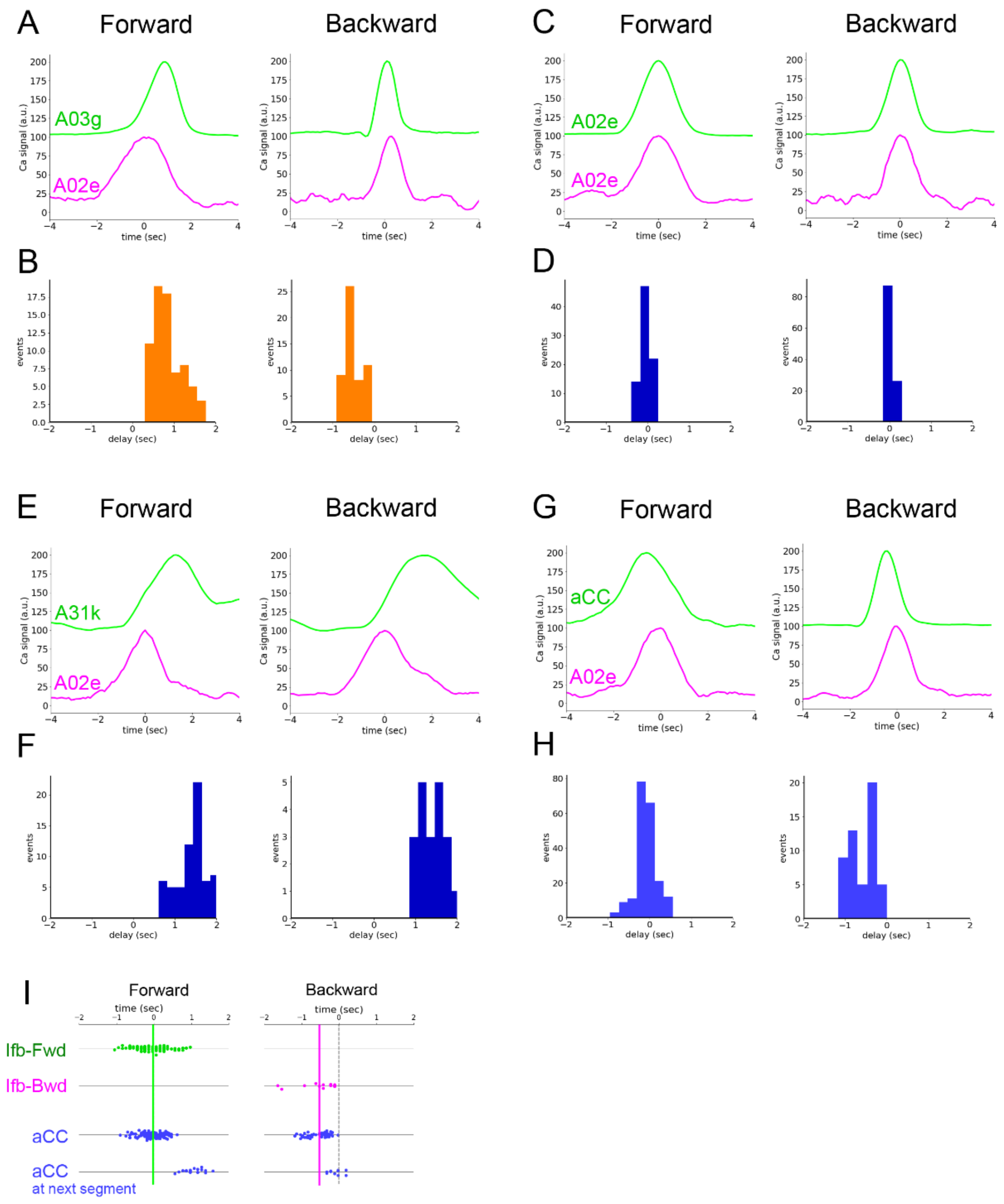

Supplementary Figure 7. Dual color calcium imaging of the shared module.
(A, C, E and G) Example traces obtained from dual color calcium imaging of A03g (A, green, n=6 larvae), A02e (C, green, n=5 larvae), A31k (E, green, n=8 larvae) and aCC MN (G, green, n=14 larvae) with A02e (magenta) as a reference during forward (left) or backward (right) waves. (B, D, F and H) Histograms of the time delay of the peak activities of A03g (B), A02e (D), A31k (F) or aCC (H) compared to A02e. (I) Scatter plot of activity peaks of aCC MNs in two neighboring segments and Ifb neurons during forward and backward waves. The timing of the activity peaks in different neurons was plotted using the A02e activity in a segment as a reference. Average activity peak of Ifb-Fwd (in forward propagation) or Ifb-Bwd (in backward propagation) was shown by a green or magenta line, respectively.

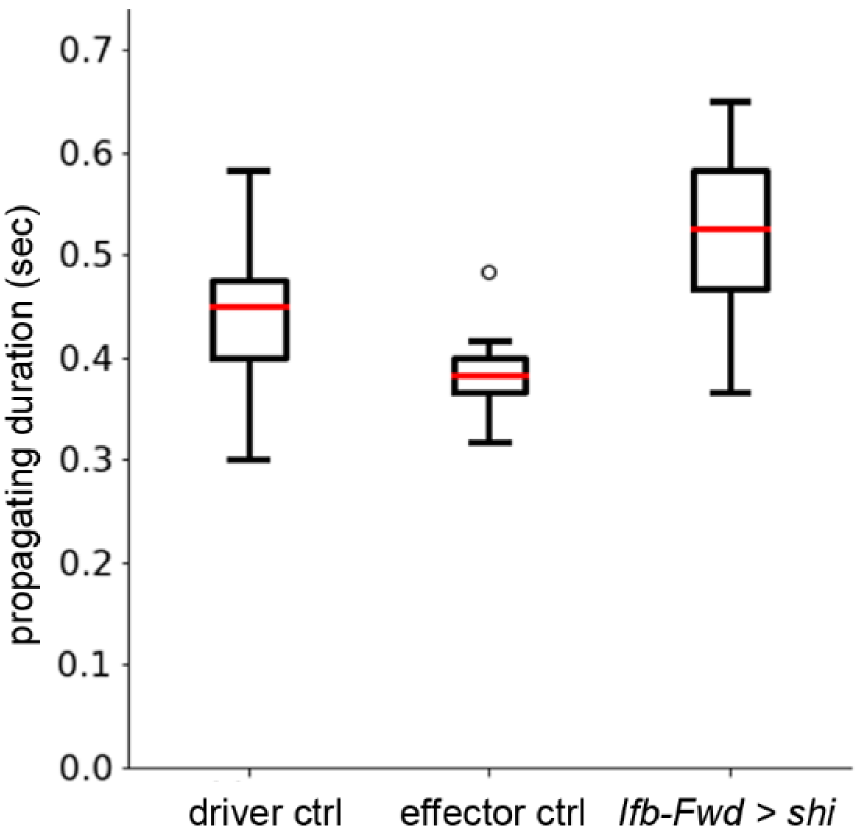

Supplementary Figure 8. Inhibition of Ifb-Fwd neurons with Shibirets decreased the speed of axial propagation of muscle contraction.

The duration for propagation of muscle contraction from the tail to head was significantly increased by inhibition of Ifb-Fwd neurons (*Ifb-Fwd>shi*, 0.51±0.01 seconds per wave (n=22); driver control, 0.44±0.02 seconds per wave (n=20); effector control, 0.38±0.01 seconds per wave (n=16), p=0.003 (driver control) and $p=8x10^{-8}$ (effector control) by the two-sided Student's t-test with the Bonferroni correction), which shows that the speed of axial propagation is decreased by inhibition of Ifb-Fwd neurons. Center line, median; box limits, upper and lower quartiles; whiskers, 1.5x interquartile range; points, outliers.

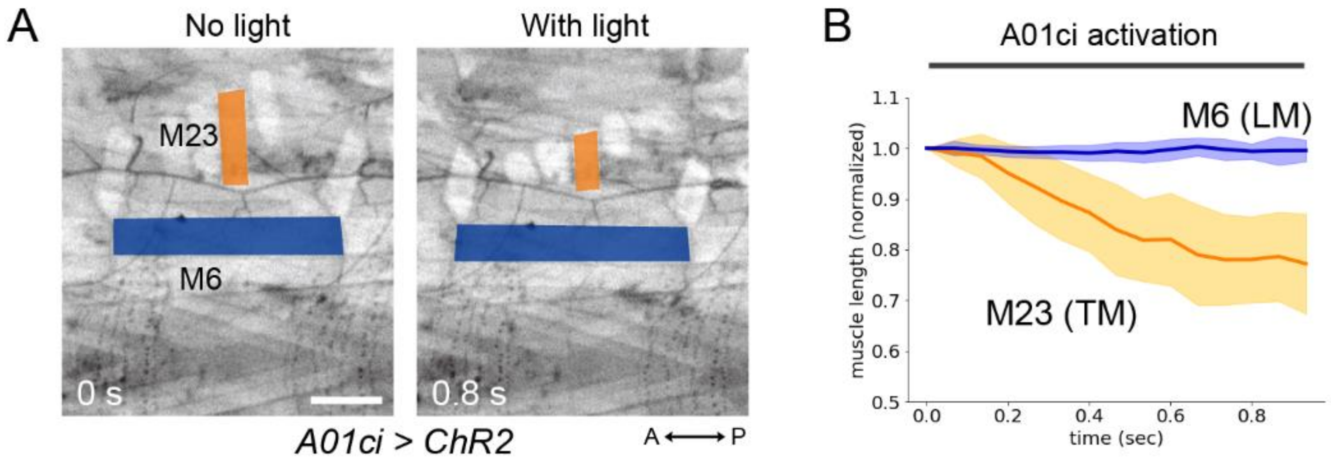

Supplementary Figure 9. Optogenetic activation of A01ci.

(A) Optogenetic activation of A01ci (by *R75H04 > ChR2*) induces contraction of a transverse but not longitudinal muscle. Muscle contraction was imaged by *mhc-GFP*. Scale bar, 100 µm. (B) Quantification of the change in muscle length upon optogenetic activation (indicated by the thick black line at the top, n=3 larvae). Solid lines indicate the mean values and transparent areas mark the standard deviation.

Supplementary References

1. Schneider-Mizell, C. M. *et al.* Quantitative neuroanatomy for connectomics in Drosophila. *Elife* **5,** (2016).

2. Burgos, A. *et al.* Nociceptive interneurons control modular motor pathways to promote escape behavior in Drosophila. *eLife* **7,** (2018).

3. Landgraf, M., Sánchez-Soriano, N., Technau, G. M., Urban, J. & Prokop, A. Charting the Drosophila neuropile: a strategy for the standardised characterisation of genetically amenable neurites. *Dev. Biol.* **260,** 207–25 (2003).
