# Supplementary material for: Regulation of forward and backward locomotion through intersegmental feedback circuits in Drosophila larvae
Source: Nat Commun. 2019 Jun 14;10:2654. doi: 10.1038/s41467-019-10695-y (PMC6572865; doi:10.1038/s41467-019-10695-y)
Supplement: Supplementary file 2 — Reporting Summary [file 41467_2019_10695_MOESM2_ESM.pdf]

nature research

Corresponding author(s): Akinao Nose

Last updated by author(s): May 23, 2019

# Reporting Summary

Nature Research wishes to improve the reproducibility of the work that we publish. This form provides structure for consistency and transparency in reporting. For further information on Nature Research policies, see Authors & Referees and the Editorial Policy Checklist .

## Statistics

For all statistical analyses, confirm that the following items are present in the figure legend, table legend, main text, or Methods section.

| n/a | Confirmed | |
|---|---|---|
| ☐ | ☒ | The exact sample size (*n*) for each experimental group/condition, given as a discrete number and unit of measurement |
| ☐ | ☒ | A statement on whether measurements were taken from distinct samples or whether the same sample was measured repeatedly |
| ☐ | ☒ | The statistical test(s) used AND whether they are one- or two-sided<br>*Only common tests should be described solely by name; describe more complex techniques in the Methods section.* |
| ☐ | ☒ | A description of all covariates tested |
| ☐ | ☒ | A description of any assumptions or corrections, such as tests of normality and adjustment for multiple comparisons |
| ☐ | ☒ | A full description of the statistical parameters including central tendency (e.g. means) or other basic estimates (e.g. regression coefficient) AND variation (e.g. standard deviation) or associated estimates of uncertainty (e.g. confidence intervals) |
| ☐ | ☒ | For null hypothesis testing, the test statistic (e.g. *F*, *t*, *r*) with confidence intervals, effect sizes, degrees of freedom and *P* value noted<br>*Give P values as exact values whenever suitable.* |
| ☒ | ☐ | For Bayesian analysis, information on the choice of priors and Markov chain Monte Carlo settings |
| ☒ | ☐ | For hierarchical and complex designs, identification of the appropriate level for tests and full reporting of outcomes |
| ☒ | ☐ | Estimates of effect sizes (e.g. Cohen's *d*, Pearson's *r*), indicating how they were calculated |

*Our web collection on statistics for biologists contains articles on many of the points above.*

## Software and code

Policy information about availability of computer code

| | |
|---|---|
| Data collection | FluoView (Olympus), iQ (Andor), VFS-42 (Sony) |
| Data analysis | Python 3.6 |

For manuscripts utilizing custom algorithms or software that are central to the research but not yet described in published literature, software must be made available to editors/reviewers. We strongly encourage code deposition in a community repository (e.g. GitHub). See the Nature Research guidelines for submitting code & software for further information.

## Data

Policy information about availability of data

All manuscripts must include a data availability statement. This statement should provide the following information, where applicable:

- Accession codes, unique identifiers, or web links for publicly available datasets
- A list of figures that have associated raw data
- A description of any restrictions on data availability

The data that support the findings of this study and the code used for the analyses are available from the corresponding authors upon reasonable request.

# Field-specific reporting

Please select the one below that is the best fit for your research. If you are not sure, read the appropriate sections before making your selection.

☒ Life sciences ☐ Behavioural & social sciences ☐ Ecological, evolutionary & environmental sciences

For a reference copy of the document with all sections, see nature.com/documents/nr-reporting-summary-flat.pdf

# Life sciences study design

All studies must disclose on these points even when the disclosure is negative.

| | |
|---|---|
| Sample size | We chose sample size by referring to our previous study (Kohsaka et al., 2014) where the difference in larval behavior was successfully quantified. |
| Data exclusions | No data were excluded. |
| Replication | We performed each experiment for multiple days and the data of each experimental group were merged into single data. Accordingly, no-repeatable factor was averaged out. |
| Randomization | We randomly picked up larvae (10 - 20) from a large population of fly larvae (more than 100) of each genotype in behavior assay. |
| Blinding | We were not blinded to group allocation. Since each experimental group consists of a distinct genotype, we didn't allocate larvae of a certain genotype into multiple groups in this study. Accordingly, we don't think we need to be blinded in this step. |

# Reporting for specific materials, systems and methods

We require information from authors about some types of materials, experimental systems and methods used in many studies. Here, indicate whether each material, system or method listed is relevant to your study. If you are not sure if a list item applies to your research, read the appropriate section before selecting a response.

## Materials & experimental systems

| n/a | Involved in the study |
|---|---|
| ☐ | ☒ Antibodies |
| ☒ | ☐ Eukaryotic cell lines |
| ☒ | ☐ Palaeontology |
| ☐ | ☒ Animals and other organisms |
| ☒ | ☐ Human research participants |
| ☒ | ☐ Clinical data |

## Methods

| n/a | Involved in the study |
|---|---|
| ☒ | ☐ ChIP-seq |
| ☒ | ☐ Flow cytometry |
| ☒ | ☐ MRI-based neuroimaging |

## Antibodies

| | |
|---|---|
| Antibodies used | rabbit or guinea pig anti-GFP (Frontier science, Af2020 and Af1180, 1:1000), mouse anti-Fas2 (DSHB #1D4, 1:10), rabbit anti-vGluT (1:1000), mouse anti-ChAT (DSHB #4B1, 1:50), rabbit anti-GABA (Sigma #A2052, 1:500), rabbit anti-DsRed (Clontech #632496, 1:500), rabbit anti-HA (Cell Signaling Technology #3724S, 1:500), Alexa488 or Cy3-conjugated goat anti-rabbit IgG, Alexa488-conjugated goat anti-guinea pig IgG and Alexa555 or Cy5-conjugated goat anti-mouse IgG (Invitrogen, 1:500). |
| Validation | anti-GFP: https://www.frontier-institute.com/wp/wp-content/uploads/pdf/GFP.pdf<br>anti-Fas2: Vactor, D., Sink, H., Fambrough, D., Tsoo, R. & Goodman, C. Genes that control neuromuscular specificity in Drosophila. Cell 73, 1137–1153 (1993).<br>anti-vGluT: Mahr, A. & Aberle, H. The expression pattern of the Drosophila vesicular glutamate transporter: a marker protein for motoneurons and glutamatergic centers in the brain. Gene Expr. Patterns 6, 299–309 (2006).<br>anti-ChAT: Takagawa, K. & Salvaterra, P. Analysis of choline acetyltransferase protein in temperature sensitive mutant flies using newly generated monoclonal antibody. Neurosci Res 24, 237–243 (1996).<br>anti-GABA: Küppers, B., Sánchez-Soriano, N., Letzkus, J., Technau, G. M. & Prokop, A. In developing Drosophila neurones the production of gamma-amino butyric acid is tightly regulated downstream of glutamate decarboxylase translation and can be influenced by calcium. Journal of neurochemistry 84, 939–51 (2003).<br>anti-DsRed: https://www.takarabio.com/assets/documents/Certificate%20of%20Analysis/632393-PA923072.pdf<br>anti-HA: https://www.cellsignal.com/products/primary-antibodies/ha-tag-c29f4-rabbit-mab/3724 |

## Animals and other organisms

Policy information about studies involving animals; ARRIVE guidelines recommended for reporting animal research

| | |
|---|---|
| Laboratory animals | Drosophila melanogaster |
| Wild animals | This study did not involve wild animals. |
| Field-collected samples | This study did not involve samples collected from the field. |
| Ethics oversight | No ethical approval was required in the study using Drosophila melanogaster. |

Note that full information on the approval of the study protocol must also be provided in the manuscript.
